# IgE-mediated cow’s milk allergy in Brazilian children: Outcomes of oral food challenge

**DOI:** 10.1016/j.waojou.2023.100781

**Published:** 2023-05-20

**Authors:** Bruna Pultrini Aquilante, Ana Paula Beltran Moschione Castro, Glauce Hiromi Yonamine, Mayra de Barros Dorna, Mariana Fernandes Barp, Tatiana Paskin da Rosa Martins, Antonio Carlos Pastorino

**Affiliations:** Allergy and Immunology Division, Pediatric Department, Instituto da Criança, Hospital das Clínicas, Faculdade de Medicina, Universidade de São Paulo (HC-FMUSP), São Paulo, SP, Brazil

**Keywords:** Milk allergy, Immunotherapy, Anaphylaxis, Epinephrine, Children, Adolescent

## Abstract

**Background:**

Oral food challenge (OFC) is useful for diagnosing food allergies and assessing tolerance, but severe reactions may occur during the procedure.

**Objective:**

To characterize the frequency and severity of reactions during cow's milk (CM) OFCs.

**Methods:**

A cross-sectional study was conducted to analyze the outcome of cow's milk oral food challenges (CMOFCs) performed to confirm IgE-mediated CM allergy or to assess food tolerance. CM was given first as baked milk (BM), followed by whole CM if there was no prior reaction to BM. An OFC was considered positive if IgE-mediated symptoms developed up to 2 h after ingestion. Symptoms were described and variables including age at OFC, prior anaphylaxis, other atopic diseases, and skin test results were compared according to the OFC outcomes.

**Results:**

A total of 266 CMOFCs were performed, including 159 patients with a median age of 6.3 years old. One hundred thirty-six tests were positive and 62 resulted in anaphylaxis. Thirty-nine anaphylactic reactions were observed up to 30 min after the first dose. Severe anaphylaxis (cardiovascular and/or neurological involvement) was reported in 5 tests. A second dose of epinephrine was required in 3 tests, and 1 presented a biphasic response. Younger patients had a higher risk of anaphylaxis during baked milk oral food challenge (BMOFC) (p = 0.009). The frequency of anaphylaxis was higher in patients submitted to BM (p = 0.009).

**Conclusions:**

Anaphylaxis is a known complication of CMOFCs even when there is no prior anaphylaxis or when conducted with baked products. This study reinforces the importance of conducting OFC in appropriate settings with a well-trained team.

## Introduction

Cow's milk allergy (CMA) is one of the most common food allergies in infants and young children,^1^ with an estimated prevalence of 0.54–3% around the world.^2^, ^3^, ^4^ In recent decades, the increased incidence of severe episodes related to CMA has concerned specialists.^5^, ^6^, ^7^ An accurate diagnosis is essential, as CMA implies dietary restrictions and special care. However, unclear clinical history and abnormal test results may often result in misdiagnosis.

For IgE-mediated CMA, careful assessment using OFC may be necessary to establish diagnosis or food tolerance. OFC, the gold standard for food allergy (FA) diagnosis, consists of a gradual feeding of the test food under close observation in an appropriate setting to treat possible allergic reactions.

Given the considerable financial and logistical constraints and risks of a severe reaction, alternative strategies to OFC have been developed. The establishment of serum IgE cut-off points and the use of molecular components^8^ have improved the diagnostic accuracy and substituted OFC in cases whose tests were previously deemed essential.^9^ Although laboratory tests can aid the diagnosis, OFC remains a precise tool to assess food tolerance when specific IgE levels decrease.

The concern of severe reactions during the OFC procedure often delays the assessment, extending the period of food restriction and negatively impacting the patients’ nutrition and quality of life. For this reason, recognizing possible outcomes during OFC can increase the safety of the procedure and encourage the medical team to perform it. Previous anaphylaxis, older age, and diagnosis of asthma are known risk factors for severe reactions during OFC^10^^,^^11^ but published data are not yet uniform, with scarce publications in Latin America. Therefore, our aim was to describe the frequency and severity of reactions during OFCs and the risk factors associated with these outcomes.

## Methods

This is a retrospective cross-sectional study based on medical records of patients who underwent CMOFC to confirm the diagnosis or evaluate food tolerance. For CMA diagnosis, all patients had either a suggestive clinical history of IgE-mediated CMA and negative IgE specific to CM and/or components or an uncertain clinical history and positive results for specific IgE to CM and/or components. For tolerance assessment, all patients had a compatible clinical history with IgE-mediated CMA and positive IgE-specific results for CM and/or components at the time of diagnosis.

All patients under 18 years old who underwent CMOFC from 2010 to 2018 at a tertiary center were included. Patients with more than 1 OFC had a record for each performed test. Patients who underwent OFC at home (regardless of the outcome) or those who presented incomplete data in the medical record and/or who did not follow the protocol standardized^12^ were excluded. All patients were admitted to a day hospital, with peripheral venous access and monitoring, under close medical supervision. Three types of OFC were performed: open; single-blind; or double-blind, placebo-controlled (DBPC). Whole CM and/or BM were tested by offering up to 300 mL or a muffin containing 2.8 g of milk protein.^12^^,^^13^

Each dose was offered within 15 to 30-min intervals with regular clinical evaluation up to 2 h after the last dose. The test was interrupted at the first sign of objective reaction and the patient was properly medicated with epinephrine, antihistamine, bronchodilators, and/or corticosteroid as indicated. A unique objective symptom initiated up to 2 h of food intake was sufficient to define a positive OFC. The standardization criteria by Sampson et al^14^ were used to define anaphylaxis.

The clinical and demographic data included were gender, age at OFC, personal atopy history (asthma, rhinitis, atopic dermatitis, other FA), and anaphylaxis. To evaluate specific IgE, a skin prick test (SPT) was performed on the day of OFC using standardized extracts (ALK-FDA Allergenic TM) of α-lactalbumin, β-lactoglobulin, casein, CM mix 5%, and whole CM (prick to prick), according to the modified Pepys technique. A positive result was defined as wheal diameter ≥3 mm compared to the negative control. For comparative analysis, the mean of 2 perpendicular wheal diameters was used.^15^^,^^16^

The statistical analysis was performed using Graphpad Prism software® (9.4.0 version, San Diego, USA). The p-value <0.05 was considered statistically significant. Nominal variables were compared using Fisher's exact test or Chi-square. Numerical or continuous variables were compared using the T-test or Mann-Whitney test as appropriate. OFC outcomes were compared according to the variables: median age at OFC, the median time to last anaphylaxis, history of anaphylaxis, diagnosis of asthma, atopic dermatitis, and median wheal diameter for CM and components in SPT.

The study was approved by the Local Research Ethics Committee (approval number 3.120.967, 2019).

## Results

Two hundred sixty-six OFCs were analyzed, including 159 patients, predominantly male (98 M: 61F), with a median age of 6.3 years old on the day of the test (1.14–17.1). One hundred six patients reported previous anaphylaxis to CM and 125 had other allergic diseases, such as rhinitis (56%), asthma (37.1%), and atopic dermatitis (22.6%). A total of 169 OFCs were performed to the whole CM and 97 to BM. Out of these 266 tests, 136 (51.1%) were positive, whereas 62 (23.3%) presented anaphylaxis.

Regarding anaphylactic outcomes, cutaneous (88.7%, 55 of 62) and respiratory (83.9%, 52 of 62) systems were the most affected (Table 1). Most OFCs (62.5%) presented early anaphylaxis (up to 30 min after the test start). There were 16 (28.6%) patients who triggered some symptoms within 10 min of the first dose, while 8 (12.5%) manifested only after the last dose. The longest time to onset of anaphylactic symptoms was 90 min after the last dose. The most commonly reported initial symptom was unspecified pruritus, which occurred after a median dose of 1/4 of a muffin (range 0.35–2.8 g of milk protein) or 50 mL of whole CM (range 1–300 mL). Both groups that reacted with or without anaphylaxis triggered symptoms from the same median dose. Cardiovascular and/or neurological involvement occurred in 5 (8.1%) OFCs that resulted in anaphylaxis. A second dose of epinephrine was required in 3 (4.8%) patients and only 1 (1.6%) of them had a biphasic response, 2 h after the first reaction. It was an eight-year-old girl diagnosed with multiple atopies and previous anaphylaxis to CM. The CM SPT had a mean diameter of 4 mm. The reaction was triggered by eating 1/8 of the muffin. After a new dose of epinephrine, the symptoms quickly resolved.

**Table 1:** Signs and symptoms of anaphylaxis in 62 CMOFCs.

Characteristics	n (%)
ANAPHYLAXIS	62 (100)
CUTANEOUS SYMPTOMS	55 (88.7)
Urticaria	33 (53.2)
Itching	30 (48.4)
Angioedema	20 (32.2)
RESPIRATORY SYMPTOMS	52 (83.9)
Cough	32 (51.6)
Bronchospasm	31 (50.0)
Rhinoconjunctivitis	23 (37.1)
GASTROINTESTINAL SYMPTOMS	37 (59.7)
Vomiting	22 (35.5)
Diarrhea	17 (27.4)
Abdominal pain	16 (25.8)
OTHER TARGET ORGAN DAMAGES	5 (8.1)
Shock	2 (3.2)
Hypotension	1 (1.6)
Sleepiness	1 (1.6)
BIPHASIC REACTION	1 (1.6)

CMOFC: cow's milk oral food challenge

A positive SPT was significantly related to a positive OFC but not related to its severity, regardless of the food tested (Table S1 and S2; supplements). The positivity of 2 or more components was also not associated with the development of anaphylaxis. There were 4 (6.4%) anaphylaxis episodes during OFC in which the SPT was negative on the day of the test.

The median age at OFC was 6.3 years, and the oldest patient was 17.1 years old. When tested for BM, younger patients had a higher risk of anaphylaxis (p = 0.009). The frequency of anaphylaxis was statistically significant according to the type of food offered. It occurred in 27 (62.8%) patients who had positive OFC to BM and in 35 (37.6%) who had positive OFC to whole CM (p = 0.009; OD, 2.79 [95% CI 1.29, 6.07]). Diagnosis of asthma was associated with anaphylaxis during OFC regardless of the food tested (p = 0.03; OD 2.09 [95% CI 1.06, 4.24]) (Table 2). Prior anaphylaxis was not related to an anaphylaxis episode during OFC, but it was significantly more frequent in positive BMOFC. (p = 0.005; odds ratio (OD), 7.17 [95% confidence interval (CI), 1.65,32.90]) Median time to last anaphylaxis and atopic dermatitis diagnosis were not statistically significant risk factors for OFC outcome or reaction severity (Table 2 and Table S3; supplements).

**Table 2:** Clinical characteristics of patients with anaphylaxis in positive OFCs.

Clinical characteristics	All OFC			WHOLE CM OFC			BM OFC		
	Anaphylaxis (n = 62)	No anaphylaxis (n = 74)	P	Anaphylaxis (n = 35)	No anaphylaxis (n = 58)	P	Anaphylaxis (n = 27)	No anaphylaxis (n = 16)	P
Median of age at OFC^a^	5.54 (0.82–17.1)	7.02 (1.1–16.8)	0.19	4.49 (0.82–15)	6 (1–17)	0.28	6.35 (4.1–17.1)	9.045 (5.2–14)	0.009^c^
Median time to last anaphylaxis (months)^a^	24 (1–126)	29 (2–127)	0.71	24 (1–121)	29 (7–127)	0.54	24 (6–126)	29 (2–48)	0.85
Prior anaphylaxis^b^	45	56	0.69	19	41	0.12	26	15	>0.99
Diagnosis of asthma^b^	32	25	0.03^d^	17	17	0.07	15	8	0.76
Diagnosis of atopic dermatitis^b^	13	17	0.83	9	13	0.80	4	4	0.44

CM: cow's milk BM: baked milk OFC: Oral food challenge.

a Mann Whitney Test.

b Fisher's Exact Test.

c 95% confidence interval of difference between medians, (0.82–3.7).

d Odds ratio, 2.09 (95% confidence interval, 1.06–4.24)

## Discussion

The OFC remains an important tool for diagnostic or tolerance assessment of food allergies. However, there are few publications that discuss the outcomes of OFC carefully and its probability to trigger anaphylaxis.^17^^,^^18^ In this FA reference center, we observed both a higher rate of allergic reactions during OFC compared to previous studies^19^, ^20^, ^21^, ^22^, ^23^, ^24^, ^25^, ^26^ and more anaphylactic events than in other studies.^19^^,^^20^^,^^25^^,^^26^ These results added up to the data of our sample previous anaphylaxis, highlighting the severity profile of a Brazilian referral center in FA.^9^

The relevance of this study in clinical practice relies on the thorough description of symptoms presented during OFC. We found out that the cutaneous system was the most affected in severe reactions, followed by respiratory, gastrointestinal, and cardiovascular systems. Few publications discuss the symptoms presented in severe reactions during OFC^10^^,^^27^^,^^28^ and the non-uniformity classification of symptoms makes a broader comparative analysis difficult. It is worth mentioning that, although most patients in our study had cutaneous involvement during anaphylaxis, the absence of these symptoms should not delay the recognition of a reaction. In addition, although subjective symptoms are not relevant for defining anaphylaxis, our study demonstrated that unspecified pruritus, rhinoconjunctivitis, or diarrhea were frequently present in anaphylactic events, demonstrating that, when persistent and evaluated together, these complaints can represent an IgE-mediated allergic reaction^29^ and be accounted as serious reactions.

Concerning the severity of anaphylactic symptoms, a smaller proportion of patients experienced cardiovascular or neurological impairment when compared to a previous similar pediatric study.^10^^,^^30^ Only 3 (4.8%) anaphylaxis episodes required more than 1 dose of epinephrine, lower than reported in the literature.^3^^,^^11^^,^^31^ These results are justified in part by promptness in epinephrine administration, facilitated by a standardized protocol application in our center.^12^ Regarding biphasic reactions, we found a similar rate compared to previous studies.^20^^,^^28^^,^^32^ Literature data reveal up to 1/3 of children with grade 4–5 anaphylaxis subsequently experience a biphasic reaction.^30^^,^^33^ In our study, only 1 patient had a biphasic response with grade 4 anaphylaxis, due to deterioration of consciousness. However, we do recognize that our small sample may limit data comparison.

Severe symptoms during OFC have been related to any step of the test.^10^^,^^34^ In our studied population, severe reactions occurred predominantly in the early steps, demonstrating the need for rigorous assistance from the first dose and a lower initial dose plan for high-risk patients. The maximum time to anaphylaxis after the last 90-min dose supports the idea that 2 h of clinical supervision from the end of the test seems safe to assure that no serious reactions occur outside the hospital. The main scope of the study was a careful description of OFC outcome, but it is important to discuss a little about specific IgE and its relationship with the test results. It is widely recognized that higher values of specific IgE are associated with a greater chance of positive OFC. There are cut-offs for specific populations which can improve the chance of a positive OFC to nearly 90%, allowing the dispensing of the test, especially for diagnosis.^35^, ^36^, ^37^, ^38^, ^39^ In contrast, establishing a cut-off for anaphylaxis is challenging and controversial. Although in 1 Japanese study,^17^ a statistically significant association was observed between anaphylaxis during OFC and higher sIgE levels to causative foods; SPT results were not able to predict anaphylaxis during OFC.

Some clinical parameters are recognized as suggestive of a serious outcome,^20^^,^^40^ but the data are still conflicting in the literature. Studies still diverge when considering age as a risk factor for anaphylaxis during OFC,^34^^,^^41^^,^^42^ possibly due to changes in the age of cow's milk tolerance in the last decades. Our study did not demonstrate statistical relevance between age at OFC and reaction risk, but it showed a higher number of anaphylaxis in younger patients tested for BM. In general, BM tests precede whole CMOFCs; therefore most serious reactions occur in younger patients. Although many patients allergic to whole milk tolerate processed foods,^43^ it is important to acknowledge potential severe reactions with this preparation form. In more recent studies, reactivity to BM has been identified as a marker of severity and persistence of CMA. Children who reacted to BMOFC experienced significantly higher rates of severe reactions and epinephrine need compared to those who tolerated BM but reacted to whole CM.^44^, ^45^, ^46^ Our result confirms this projection since the proportion of anaphylaxis triggered in BMOFC was significantly higher than in whole CM tests. It is noteworthy that all CMOFCs were either preceded by a successful BMOFC or just performed with no previous test.

Considering other risk factors for anaphylaxis during OFC, a history of prior anaphylaxis shows divergent results in the literature.^10^^,^^47^ In our sample, prior anaphylaxis was not a risk factor for anaphylaxis during OFC. Indeed, our study showcased a high rate of prior anaphylaxis for few anaphylactic outcomes. This may reflect a precise choice for the best time to test, based on falling values of specific IgE and past accidental ingestion without reaction. Therefore, we emphasize that an isolated past medical should not discourage a team to perform the procedure. It is necessary to consider the natural history of each food allergen to decide the best moment to evaluate tolerance. In our study part of the challenges were performed to document the development of tolerance. Some predictors of tolerance onset are inadvertent ingestion of food in small amounts with mild or absent manifestations or a decrease of specific IgE. Conversely, it is important to be aware of the risk of anaphylaxis in patients that had never had a history of anaphylaxis to culprit food. Our study demonstrated that 17 (27.4%) patients who developed anaphylaxis during OFC had never experienced anaphylaxis. The absence of prior anaphylaxis does not define a patient as low-risk; in fact, it may only represent a situation in which past intakes were insufficient to trigger this reaction.

Admittedly, asthma is a risk factor for severe reactions during OFC due to a lower available physiological reserve and greater inflammatory activity concomitant with the allergic process.^17^^,^^48^^,^^49^ In our analysis, asthma was also considered a risk factor for anaphylaxis in OFC, regardless of the food tested.

One study limitation was the low rate of OFC performed as DBPC. Our choice was due to methodological difficulties, time, and financial limitations. Literature data refers to a positive rate of up to 2.8% in placebo tests,^50^ but the use of a standardized protocol and the description of objective outcomes with anaphylaxis allows the data to be validated. Another limitation of the study was that some patients did not perform BM before the whole CM, and the main reason was that some patients were too young to accept this preparation.

## Conclusion

Although OFC is recognized as the gold standard for FA diagnosis, clinicians and researchers admit concern about the risk of exposing the patient to a severe systemic reaction. Some clinical trials with allergic patients developed predictive models for anaphylaxis risk stratification during OFC,^44^^,^^51^^,^^52^ but further studies are still needed. Our sample was able to point out the relevance of the BM step and its inherent risks. We also observed the presence of anaphylaxis during OFC even with no history of past anaphylaxis. Customizing the best time for OFC to achieve favorable results still requires further analysis.

Even though FA diagnosis has improved with recent advances involving molecular components, OFC still remains the most reliable procedure for confirming the diagnosis and assessing tolerance. Staff training to treat serious reactions regardless of the type of preparation offered, along with proper emergency care, can minimize complications and risks.

## Abbreviations

OFC, oral food challenge; CM, cow's milk; BM, baked milk; CMOFC, cow's milk oral food challenge; BMOFC, baked milk oral food challenge; CMA, cow's milk allergy; FA, food allergy; DBPC, double-blind, placebo-controlled; SPT, skin prick test.

## Acknowledgments

We thank the Reviewers for their critical contributions to this manuscript.

## Funding

This project did not receive any specific funding from any agency, commercial or not not-for-profit sectors.

## Availability of data and materials

Please contact corresponding author for primary data requests.

## Author’ contributions

BPA, ACP, and APBMC conceived and coordinated the study and participated in its design. BPA prepared the first draft and performed the clinical approach. MFB and TPRM and GHY performed in vivo and food oral challenges tests. BPA performed the statistical analysis and translated the manuscript. MFB, MBD, and TPRM helped and reviewed to draft of the manuscript. All authors read and approved the final version of this manuscript.

## Ethical aspects

The study was approved by the Research Ethics Committee of Hospital das Clínicas- USP, São Paulo, Brazil (approval number 3.120.967, 2019). The local Ethics Committee reviewed our study and determined it was exempt from requiring ethics approval or subject consent, as it did not meet the definition of “human subject” research.

## Consent for publication

All authors made intellectual contributions during the drafting and revision of the work, approved the final version to be published, and agreed to be accountable for all aspects of the work, including accuracy and integrity. All authors gave their written consent for publication.

## Declaration of competing interests

The authors declare no conflict of interest related to this study.
