# Supplementary material for: IgE-mediated cow’s milk allergy in Brazilian children: Outcomes of oral food challenge
Source: World Allergy Organ J. 2023 May 20;16(5):100781. doi: 10.1016/j.waojou.2023.100781 (PMC10209878; doi:10.1016/j.waojou.2023.100781)
Supplement: Multimedia component 1 [file mmc1.pdf]

Supplemental Table 1

Table S1. Skin prick test in patients with positive and negative OFC for whole CM and BM

Wheal diameters median in mm at SPT	WHOLE CM OFC				BM OFC			
	positive (n=93)	negative (n=76)	p*	(95% CI)**	positive (n=43)	negative (n=54)	p*	(95% CI)**
Whole CM	4.5 (0-25)	1 (0-10)	< 0.0001	(1.5,4.0)	6 (0-18)	4.5 (0-10.5)	0.06	(0.0,4.0)
Mix CM 5%	3 (0-16)	1.5 (0-14.5)	0.006	(0.0,2.0)	4.5 (0-12.5)	3 (0-8.5)	0.08	(0.0,3.0)
Casein	3.25 (0-11.5)	1 (0-18.5)	< 0.0001	(1.0,2.5)	5.5 (0-13.5)	3 (0-9.5)	0.02	(0.0,3.0)
α-lactalbumin	5 (0-17)	2 (0-14)	0.01	(0.0,3.0)	7.5 (0-18)	4.5 (0-13)	0.002	(1.0,4.5)
β-lactoglobulin	5 (0-13.5)	2 (0-11.5)	0.001	(0.5,3.5)	5.5 (0-18)	4.5 (0-15)	0.61	(-1.5,2.5)

CM: cow's milk BM: baked milk OFC: Oral food challenge SPT: skin prick test

*Mann Whitney Test
**95% confidence interval of difference between medians

Supplemental Table 2

Table S2. Skin prick test in patients with anaphylaxis during OFC

Wheal diameters median in mm at SPT	All OFC				WHOLE CM OFC				BM OFC			
	Anaphylaxis (n=62)	No anaphylaxis (n=74)	P*	(95% CI)**	Anaphylaxis (n=35)	No anaphylaxis (n=58)	P*	(95% CI)**	Anaphylaxis (n=27)	No anaphylaxis (n=16)	P*	(95% CI)**
Whole CM	6 (0-18)	4.5 (0-25)	0.33	(-1.0,2.0)	5.5 (0-12)	4.5 (0-25)	0.43	(-1.0,2.5)	6 (0-18)	6 (0-14)	0.96	(-4.0,4.0)
Mix CM 5%	4 (0-12.5)	3.5 (0-16)	0.54	(-1.0,0.5)	3 (0-10.5)	3 (0-16)	0.82	(-1.0,0.5)	4.5 (0-12.5)	4 (0-8.5)	0.60	(-2.0,4.0)
Casein	4.25 (11.5)	3.5 (0.13.5)	0.33	(-0.5,1.5)	3.75 (0-11.5)	3 (0-9.5)	0.34	(-0.5,2.0)	5.25 (0-11)	5.5 (0-13.5)	0.84	(-3.0,2.0)
α-lactalbumin	5.75 (0-18)	5.5 (0-12.5)	0.72	(-2.0,1.0)	5 (0-17)	5 (0-12.5)	0.59	(-2.0,1.0)	6.5 (0-18)	9 (0-12.5)	0.13	(-5.0,0.5)
β-lactoglobulin	5.5 (0-18)	4.5 (0-11)	0.20	(-2.5,0.0)	6 (0-13.5)	4 (0-10.5)	0.05	(0.0,3.5)	5 (0-18)	6.5 (0-9)	0.51	(-3.0,2.0)

CM: cow's milk BM: baked milk OFC: Oral food challenge

*Mann Whitney Test
**95% confidence interval of difference between medians

Supplemental Table 3

Table S3. Clinical characteristics of patients undergoing whole CM and BM OFC

Clinical characteristics	WHOLE CM OFC			BM OFC		
	positive (n=93)	negative (n=76)	p	positive (n=43)	negative (n=54)	p
Median of age at OFC*	5.49 (0.82-16.8)	5.15 (1-15.3)	0.82	7.94 (4.1-17.1)	6.72 (1.6-14.2)	0.42
Median time to last anaphylaxis (months)*	29 (1-127)	31 (5-87)	0.26	24 (2-126)	31 (10-127)	0.19
Prior anaphylaxis**	60	45	0.52	41	40	0.005***
Diagnosis of asthma**	34	18	0.09	23	23	0.31
Diagnosis of atopic dermatitis**	22	18	0.99	8	11	0.99

CM: cow's milk BM: baked milk OFC: Oral food challenge

*Mann Whitney Test
**Fisher's Exact Test
*** Odds ratio, 7.17 (95% confidence interval, 1.65-32.90)
